# Machine learning based gut microbiota pattern and response to fiber as a diagnostic tool for chronic inflammatory diseases

**DOI:** 10.1186/s12866-025-04072-7

**Published:** 2025-06-06

**Authors:** Miad Boodaghidizaji, Thaisa Jungles, Tingting Chen, Bin Zhang, Tianming Yao, Alan Landay, Ali Keshavarzian, Bruce Hamaker, Arezoo Ardekani

**Affiliations:** 1 School of Mechanical Engineering, Purdue University, 585 Purdue Mall, West Lafayette, IN 47907 USA; 2 Department of Food Science, Whistler Center for Carbohydrate Research, Purdue University, West Lafayette, IN 47907 USA; 3 Departments of Internal Medicine, Anatomy and cell biology, and Molecular Biophysics and Physiology, Center for Integrated Microbiome and Chronobiology Research, Rush University Medical Center, Chicago, USA; 4 State Key Laboratory of Food Science & Technology, Nanchang University, Nanchang, China; 5 School of Food Science and Engineering, South China University of Technology, Guangzhou, 510640 China

**Keywords:** Microbiome data, Machine learning, Ulcerative colitis, Crohn's disease, Fiber treatment

## Abstract

Gut microbiota has been implicated in the pathogenesis of multiple gastrointestinal (GI) and systemic metabolic and inflammatory disorders where disrupted gut microbiota composition and function (dysbiosis) has been found in multiple studies. Thus, human microbiome data holds significant potential as a source of information for diagnosing and characterizing diseases—such as phenotypes, disease course, and therapeutic response—associated with dysbiotic microbiota communities. However, multiple attempts to leverage gut microbiota taxonomic data for diagnostic and disease characterization have failed due to significant inter-individual variability of microbiota community and overlap of disrupted microbiota communities among multiple diseases. One potential approach is to look at the microbiota community pattern and response to microbiota modifiers like dietary fiber in different disease states. This approach has become feasible with the advent of machine learning, which can uncover hidden patterns in human microbiome data and enable disease prediction. Accordingly, the aim of our study was to test the hypothesis that machine learning algorithms can distinguish stool microbiota patterns—and their responses to fiber—across diseases with previously reported overlapping dysbiotic microbiota profiles. Here, we applied machine learning algorithms to distinguish between Parkinson's disease, Crohn's disease (CD), ulcerative colitis (UC), human immune deficiency virus (HIV), and healthy control (HC) subjects in the presence and absence of fiber treatments. We demonstrated that machine learning algorithms can classify diseases with accuracy as high as 95%. Furthermore, applying machine learning to microbiome data to distinguish UC from CD yielded a prediction accuracy of up to 90%.

**Supplementary Information:**

The online version contains supplementary material available at 10.1186/s12866-025-04072-7.

## Background

Microbial communities play invaluable functional roles in supporting human health, influencing immune function, metabolism, brain activity, and behavioral traits [1]. The human gastrointestinal tract harbors the largest population of micro-organism community, known as gut microbiota. This community is typically composed of a variety of micro-organisms, including bacteria, archaea, and eukarya [2]. The gut microbiota actively participates in human metabolism—contributing to the synthesis of vitamins and other nutrients, regulating immune functions, and promoting gut barrier integrity, among other roles [3]. Not surprisingly, several disease-related states have been linked to imbalances in the gut microbial community, a phenomenon known as dysbiosis [4]. Whether gut microbiota dysbiosis is a cause, consequence, or both in different disease states, it contains invaluable information that may aid in disease diagnosis. However, due to the high dimensionality of microbiome data, important patterns may remain undetected using traditional analytical methods. 

A notable challenge is the variation of the gut microbiota across individuals. This variation is influenced not only by disease states but also by genetic background and several environmental factors, including diet and dietary fiber intake, which differ both between and within individuals and populational groups [5]. Such variability complicates efforts to diagnose diseases based on clustering through commonly used beta diversity ordination plots, which often show overlapping patterns across health and disease states, making group separation unclear [6–8].

Recently, Machine learning (ML) methods have opened new avenues for exploring of gut microbiota data in ways previously unattainable. Supervised and unsupervised machine learning methods have been employed for classification, regression, clustering, and non-negative matrix factorization [9]. These methods have been successfully implemented to distinguish healthy subjects from those with gastrointestinal (GI) diseases, such as inflammatory bowel diseases (IBD) [10]. Furthermore, ML models have shown promising results in predicting diseases that do not directly affect the GI tract, such as cardiovascular diseases [11].

Although there is a myriad of studies that have applied ML to predict disease states from gut microbiota, most have focused on distinguishing healthy from non-healthy subjects or predicting diseases that fall within similar categories, such as different GI disease, including IBD and esophageal diseases [12, 13]. As a result, it remains critical to assess whether the predictive capabilities of ML models are limited to certain classes of diseases or whether they can be extended to simultaneously classify multiple GI and systemic diseases that share overlapping dysbiotic microbiota profiles. For example, previous research has reported similarities in the gut microbiota of patients with Parkinson’s disease and those with IBD [14]. Additionally, it is unclear whether these diseases can still be distinguished after treatment—especially after fiber-based interventions. To address these questions, in the current study, we aim to apply machine learning techniques to classify five different conditions: Parkinson's disease (PD), Crohn's disease (CD), ulcerative colitis (UC), human immune deficiency virus (HIV), and healthy control (HC) subjects both in the presence and absence of fiber treatments.

## Materials and methods

### Materials

Fecal samples from 10 healthy individuals (HC), 10 Parkinson’s disease patients (PD), 7 inactive Crohn’s disease patients (CD), 7 inactive ulcerative colitis patients (UC), and 2 HIV patients (HIV) were received from Rush University Medical Center. For each individual, we acquired multiple reads corresponding to various types of treatments, significantly expanding the pool of available unique cases to a total of 1094. The samples were frozen at −80 °C and shipped overnight with dry ice. Five fermentable soluble dietary fibers commonly present in diets were used for the study. Fructooligosaccharides from sugar cane (Nutraflora, Ingredion, USA), barley beta-glucan (P -BGBM, Megazyme, Bray, Ireland), apple pectin (AF 710, Herbstreith&Fox Inc., Germany), sorghum arabinoxylan (extracted as previously described, Rumpagaporn et al., 2015 [15]), and a mixture of the four (fructooligosaccharides, beta-glucan, pectin and arabinoxylan, 25% each). We selected these disease groups because prior studies have shown that they all are associated with dysbiotic microbiota characterized by a relative abundance of “pro-inflammatory” bacteria, like bacteria belonging to protobacter phyla, and decreased relative abundance of “anti-inflammatory” bacteria, including short-chain fatty acids (SCFA) producing bacteria. Thus, there was an overlap in changes in their microbiota community that resulted in difficulty in using microbiota data as a diagnostic and/or disease prediction tool.

### In vitro fecal fermentation

The fecal samples were thawed in the anaerobic chamber 30 min prior to the in vitro fecal fermentation experiment. The fermentation procedure used was similar to that described by Kaur et al., 2011 [16], except that all the procedures were conducted in an anaerobic chamber instead of using CO_2_ flushing. Dietary fiber (1%) and 5% feces were added to 5 mL of PBS buffer in an anaerobic tube. The tubes were sealed and incubated at 37 °C for 12 hours. After fermentation, the fermenta were collected and centrifuged at 14,000 g for 5 minutes, and DNA was extracted from the pellet using FastDNA™ SPIN Kit for feces (116,570,200, MP Biomedicals, USA). Informed consent was obtained from all donors, and experiments were approved by the ethical committee at Purdue University (IRB 1509016451). Further, all participants signed the Rush University Medical Center (RUMC) Institutional Review Board approved informed consent forms (ORA#: 07100403; 12,020,204; 07092603; L04092807).

### DNA sequencing and data preprocessing

The DNA obtained before and after in vitro fecal fermentation of stools was analyzed by 16S ribosomal RNA (rRNA) sequencing, which was performed by the DNA Services Facility at the University of Illinois at Chicago. Briefly, the V3-V4 region of the extracted DNA was amplified using the 341F/806R primer set. The amplicon was detected by agarose gel electrophoresis. A second polymerase chain reaction (PCR) analysis was performed on the common sequences with primers containing Illumina adapters, a sample‐specific barcode (10 bases), and linker sequences (called common sequences) at the ends of the forward and reverse primers. After 2 stages of PCR, the amplicons were sequenced using an Illumina MiSeq sequencer. The obtained raw sequences, which contain forward R1 and reverse R2, were merged. Chimeras were removed using the USEARCH algorithm, and sequences were then merged into one FASTA file and subject to the UPARSE pipeline for operational taxonomic unit (OTU) clustering [17]. The taxonomic information for each OTU was determined using a ribosomal database project (RDP) classifier [18]. For the preprocessing, the OTU based data was normalized with respect to the cumulative count of all the microbiota for each subject before feeding into ML models. Then, the genus level data was used for ML analysis.

### Machine learning modeling

For all the classification tasks in this study, we employed four different ML algorithms: random forest (RF), support vector machine (SVM), artificial neural networks (ANN), and convolutional neural network (CNN). These methods have been successfully implemented to solve many problems that involve genomic datasets. [12]. All of these algorithms can be utilized for binary and multi-class classification purposes, such as distinguishing healthy vs. non-healthy and healthy vs. Parkinson's disease vs. colitis, respectively. To implement SVM and RF, we used scikit-learn classifiers [19] in python, where the one-vs-one scheme is used for multi-label classification. To implement ANN and CNN, the Multi-Layer Perceptron (MLP) classifier of scikit-learn [19] and PyTorch [20] were used, respectively. Additionally, to prevent unbiased comparison, the same preprocessed data was fed to each ML model. To find the best model parameters and compare different machine learning models, we used a fivefold cross-validation technique, where we used the average values of the predictions of all the 5 folds to report classification metrics. We reported commonly used classification performance metrics, including the macro and micro F1 scores, recall, precision, and accuracy. Micro-averaged metrics reflect averages over all instances, while macro-averaged metrics represent unweighted averages across all classes [21].

In SVM, a hyperplane is constructed to separate data points with the largest possible margin For a given kernel function, SVM identifies an optimal hyperplane that classifies the data into distinct groups. In general, SVMs work well when a clear margin of separation exists, and they can efficiently learn complex classification functions and employ powerful regularization principles to avoid data over-fitting [12]. SVM has shown promising performance in classifying healthy vs. non-healthy subjects in various domains including lung cancer [22] and obesity [23]. In this study, we used an SVM with a non-linear radial basis functions (RBF) kernel.

RF utilizes an ensemble average of multiple decision trees, each trained on a bootstrap sample of the dataset. For classification tasks, RF predicts the class label by aggregating the majority vote from individual trees. This ensemble approach paves the way for learning both complex and simple functions. An additional advantage of RF is its ability to estimate feature importance, which is used for selecting the most informative variables. One of the primary strengths of RF is the capability to handle datasets with a large number of predictor variables [24]. Furthermore, in general, RF does not require a comprehensive grid search for hyper-parameter optimization, and the default parameters lead to acceptable accuracy [12]. In microbiome research, RF has been successfully applied for disease classification tasks, including bipolar disorders [25], coronary artery disease [26], and major depressive disorder [27]. In the current study, the RF model was configured with 100 trees, maximum depth of 10, and used the Gini impurity criterion to evaluate the quality of split.

**Fig. 1:**
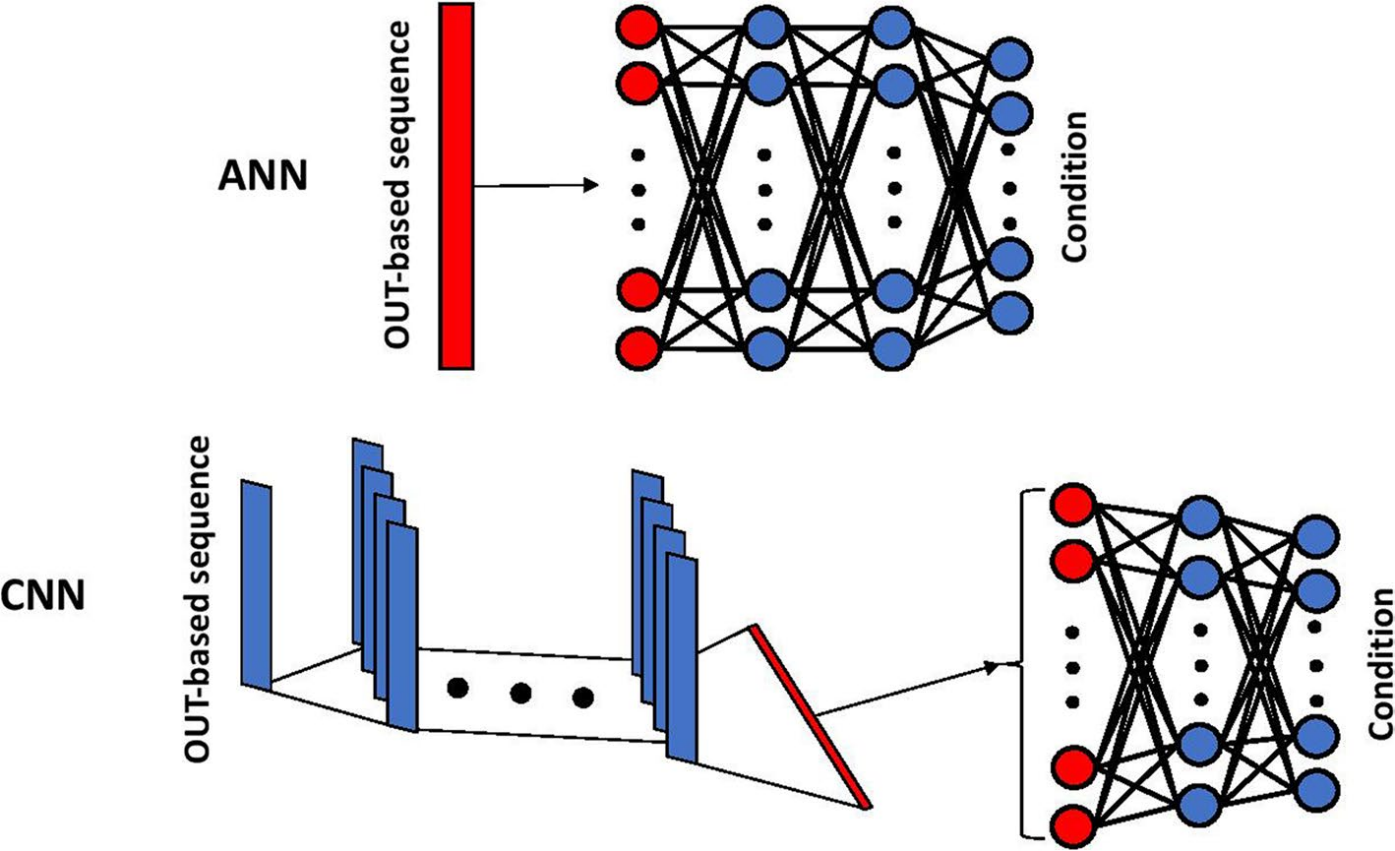
Schematic representation of the ANN and CNN architecture used to predict the patient’s conditions. The number of neurons and layers shown here are for illustrative purposes and do not represent the exact configurations used in the analysis

 Inspired by biological neural networks, deep learning methods such as ANN and CNN consist of multiple hidden layers and numerous neurons, enabling them to tackle a wide range of problems. Unlike most traditional ML methods, neural networks incorporates a built-in feature selection mechanism by assigning weights to input features and applying activation functions, thereby learning the importance of each feature during training. Further, in the case of CNN, applying convolutional layers enables the detection of spatial, and dependencies in the input signals. Both CNN and ANN have been applied to different classification problems involving gut microbiota, such as obesity [28], inflammatory bowel disease [29], and Parkinson's disease [30] detection. Various adaptation of CNNs with different levels of data preprocessing, such as Met2Img and Metal ML, which involve data augmentation and feature extraction strategies, have been formed and applied to gut microbiota data [31]. Here, after preprocessing and arranging the data into OTU format, we directly input the data into ANN and CNN models. The architecture used for both CNN and ANN are shown in Fig. 1.We used the cross-entropy loss function for both models, with outputs representing class probabilities.

## Results and discussions

**Fig. 2:**
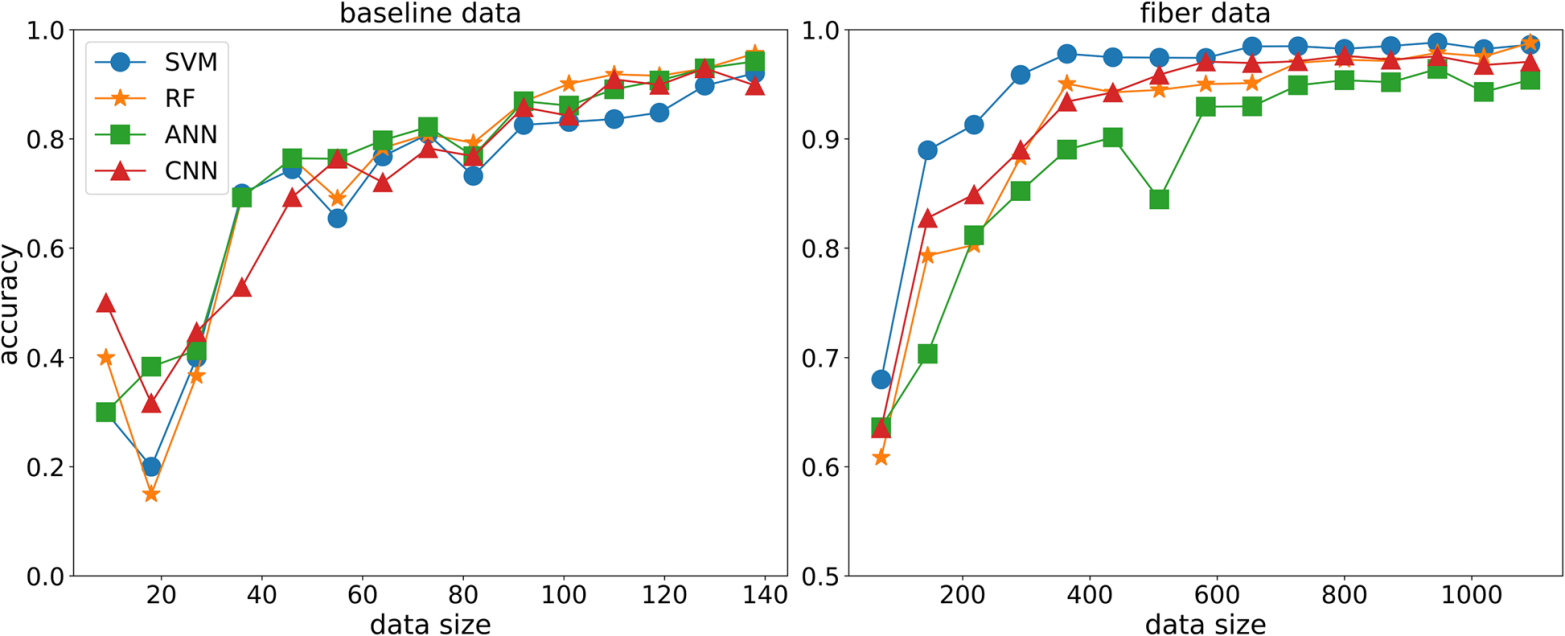
Prediction accuracy as a function of data size for the baseline and fiber datasets

We used two different datasets: the first dataset included samples without any treatment; the second dataset comprises samples with and without fiber treatment. Throughout the manuscript, we refer to the first and second datasets as"baseline"and"fiber"datasets, respectively. For both datasets, we varied the number of data points and evaluated how classification accuracy changed as a function of dataset size, as shown in Fig. 2. In both cases, the prediction accuracy increased with the data size and exceeded 95%. For classification, we used the maximum data size, where 138 and 1092 data points were available for the baseline and fiber datasets, respectively. Additionally, since all micro-averaged metrics—including micro F1 score, recall, precision, and accuracy—had identical values across our experiments, we only reported accuracy as representative of all the micro metrics.

### Prediction of the diseases based on the baseline dataset

**Fig. 3:**
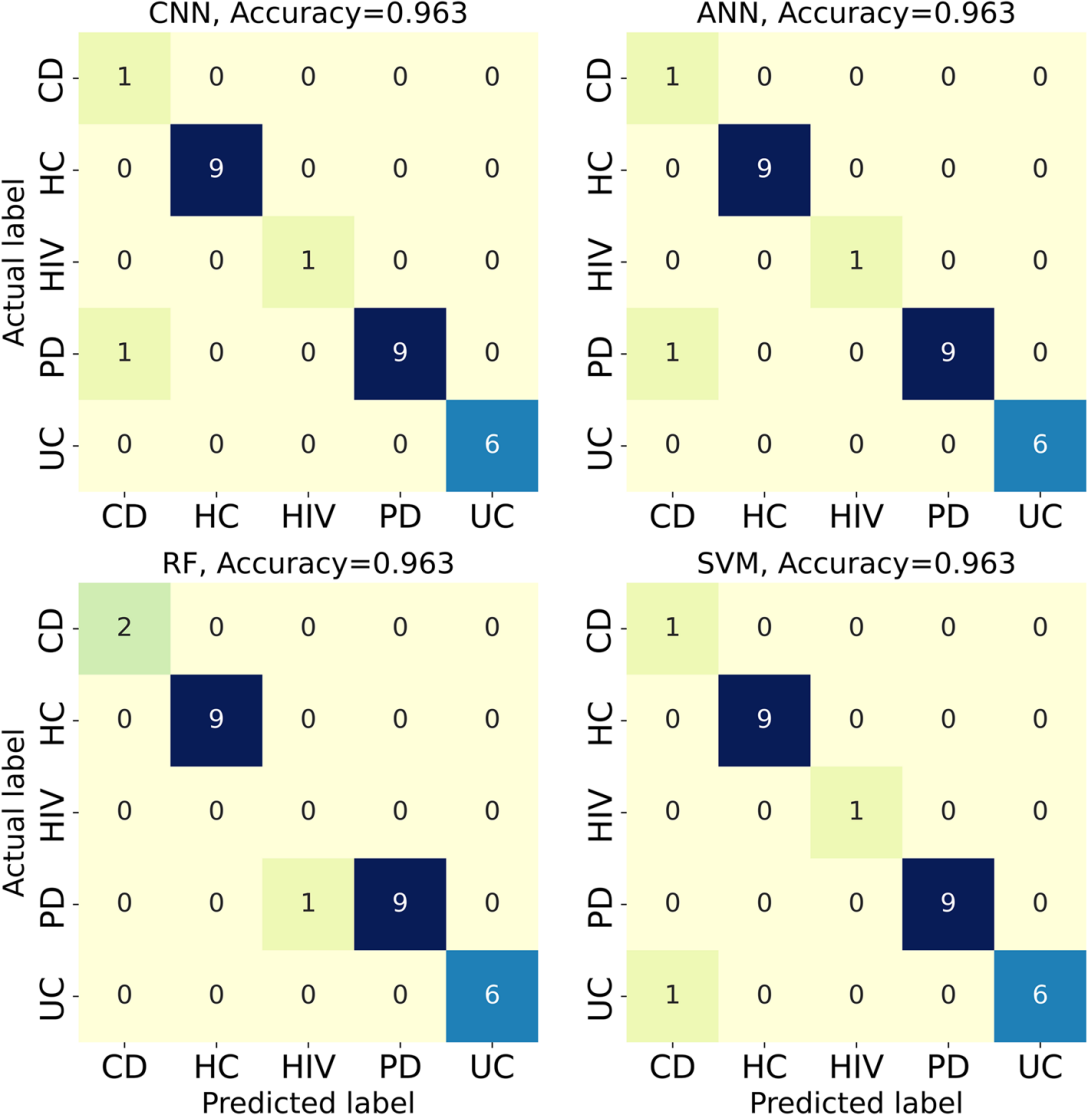
Confusion matrices for the classification of the five conditions using the baseline dataset

**Fig. 4:**
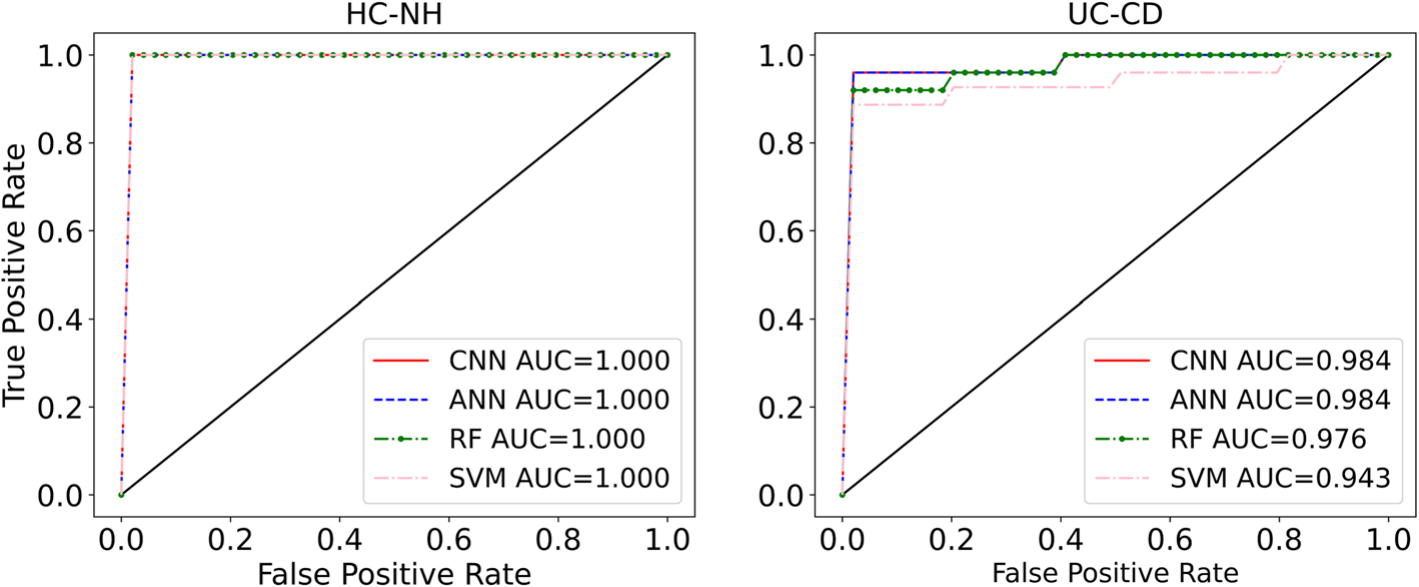
ROC curves with AUC values listed for the binary classification of HC vs. NH and UC vs. CD using the baseline dataset

Here, we present the results for three classification tasks: 1) the five conditions, 2) the HC vs. non-healthy (NH) 3), and the UC vs. CD. Table 1 demonstrates the prediction performances for the baseline dataset. The results indicate that SVM achieves the highest values across all classification metrics. However, the difference between the algorithms is not significant. The heatmap for the classification of the five conditions for one of the representitive folds of the fivefold cross-validation is shown in Fig. 3. We note that algorithms have difficulty identifying UC vs. CD and HIV vs. PD diseases. The data points corresponding to HIV cases are relatively low in the dataset compared to other conditions, which contributes to the inability of the algorithms to detect HIV cases. To demonstrate that increasing HIV cases resolves the misclassification of HIV cases, we have augmented the baseline data with microbiota data obtained from Mutlut et al. [32] for HIV patients, as demonstrated in supplementary section A. For UC vs. CD classification, the classification metrics revealed that the models are not able to perfectly distinguish between the two, which is in line with what previous machine learning studies suggest. Indeed, despite attempts using diverse data modalities, such as RNA sequencing data [33] and endoscopic images [34], no method has yet achieved perfect UC vs. CD discrimination. Additionally, we note that in the case of HC and NH, all the accuracies are relatively high across all methods, highlighting the strong capability of ML methods to distinguish between HC and NH. As shown in Table 1, the ML models models demonstrated near-perfect classification, where the macro and micro metrics reached as high as 99%. In other words, ML algorithms can perfectly identify the trend distinguishing HC and NH cases, which is further reflected in AUC values, as shown in Fig. 4. Furthermore, we conducted dimensionality reduction through random projection and principal component analysis (PCA) and repeated the classification tasks. Comparable prediction performance was observed for the five conditions, as demonstrated in supplementary section B.

**Table 1:** Classification performances using the baseline data

**Task**	**Classifier**	Macro precision	Macro Recall	Macro F1	Micro	Accuracy
Five conditions	RF	0.890 $\pm$ 0.113	0.888 $\pm$ 0.103	0.885 $\pm$ 0.110	0.957 $\pm$ 0.035	0.957 $\pm$ 0.035
	SVM	0.975 $\pm$ 0.022	0.947 $\pm$ 0.051	0.952 $\pm$ 0.045	0.971 $\pm$ 0.027	0.971 $\pm$ 0.027
	ANN	0.953 $\pm$ 0.034	0.937 $\pm$ 0.036	0.940 $\pm$ 0.033	0.949 $\pm$ 0.029	0.949 $\pm$ 0.029
	CNN	0.958 $\pm$ 0.035	0.942 $\pm$ 0.039	0.946 $\pm$ 0.035	0.957 $\pm$ 0.027	0.957 $\pm$ 0.027
HC-NH	RF	0.995 $\pm$ 0.011	0.989 $\pm$ 0.022	0.991 $\pm$ 0.017	0.993 $\pm$ 0.015	0.993 $\pm$ 0.015
	SVM	1.000 $\pm$ 0.000	1.000 $\pm$ 0.000	1.000 $\pm$ 0.000	1.000 $\pm$ 0.000	1.000 $\pm$ 0.000
	ANN	1.000 $\pm$ 0.000	1.000 $\pm$ 0.000	1.000 $\pm$ 0.000	1.000 $\pm$ 0.000	1.000 $\pm$ 0.000
	CNN	1.000 $\pm$ 0.000	1.000 $\pm$ 0.000	1.000 $\pm$ 0.000	1.000 $\pm$ 0.000	1.000 $\pm$ 0.000
UC-CD	RF	0.892 $\pm$ 0.047	0.863 $\pm$ 0.045	0.865 $\pm$ 0.051	0.869 $\pm$ 0.049	0.869 $\pm$ 0.049
	SVM	0.913 $\pm$ 0.049	0.887 $\pm$ 0.077	0.883 $\pm$ 0.077	0.887 $\pm$ 0.072	0.887 $\pm$ 0.072
	ANN	0.874 $\pm$ 0.101	0.867 $\pm$ 0.102	0.866 $\pm$ 0.102	0.867 $\pm$ 0.101	0.867 $\pm$ 0.101
	CNN	0.860 $\pm$ 0.087	0.847 $\pm$ 0.081	0.847 $\pm$ 0.083	0.849 $\pm$ 0.082	0.849 $\pm$ 0.082

### Prediction of the diseases based on the fiber dataset

**Fig. 5:**
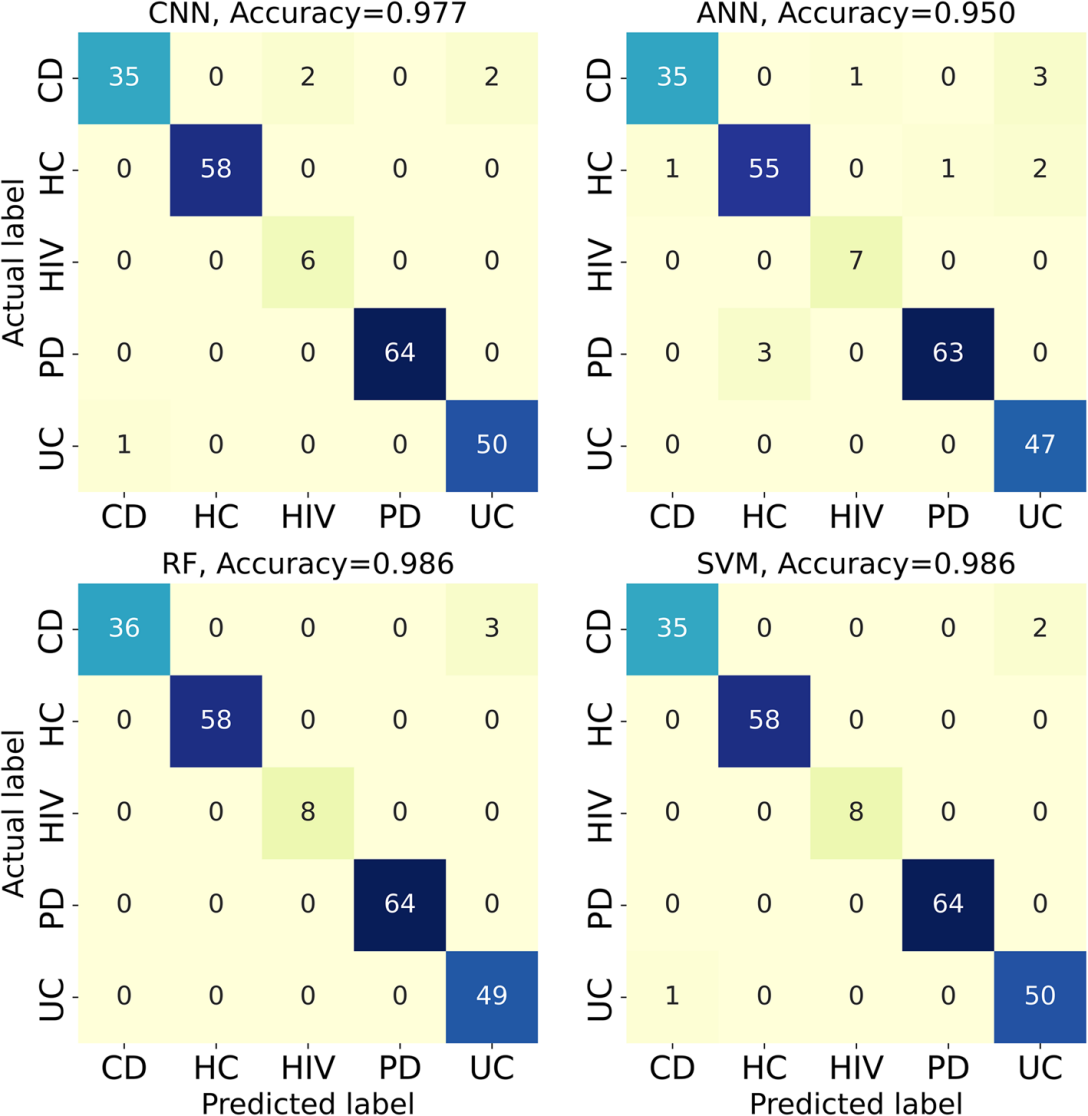
Confusion matrices for the classification of the five conditions using the fiber dataset

As with the baseline dataset, we performed three classification taks for the fiber dataset. Dietary fiber is known to modify the gut microbiota composition [35], adding another layer of intraindividual gut microbiota variability that could help with the classification of individuals amongst disease states. Table 2 demonstrates how the classification metrics vary for different algorithms when fiber data is introduced. We notice that RF and SVM methods lead to predictions with higher accuracy compared to ANN and CNN. Furthermore, the increased number of data points in the fiber data outweighs the microbial shifts caused by dietary fiber and significantly boosts the classification accuracy—particularly in differentiating the five individual conditions. As shown in Fig. 5, which illustrates the heatmap for one of the representitive folds of fivefold cross-validation, most misclassified conditions belong to UC, CD, and HIV cases. Further, we observe that for the UC vs. CD classification, increasing the data size improved the classification accuracy compared to the baseline dataset. However, the algorithms still misidentify a small number of the CD cases as UC and vice versa (3 out of 88, Fig. 5). These results are further supported by the receiver operating characteristic curve (ROC) curves and area under characteristic curve (AUC) values (Fig. 4), where prediction accuracy for UC vs. CD reached as high as 97% (RF, Table 2).

**Table 2:** Classification performances using the fiber dataset

**Task**	**Classifier**	Macro precision	Macro Recall	Macro F1	Micro	Accuracy
Five conditions	RF	0.989 $\pm$ 0.003	0.990 $\pm$ 0.005	0.990 $\pm$ 0.004	0.989 $\pm$ 0.005	0.989 $\pm$ 0.005
	SVM	0.990 $\pm$ 0.004	0.985 $\pm$ 0.007	0.987 $\pm$ 0.005	0.988 $\pm$ 0.005	0.988 $\pm$ 0.005
	ANN	0.934 $\pm$ 0.037	0.947 $\pm$ 0.028	0.934 $\pm$ 0.028	0.958 $\pm$ 0.018	0.958 $\pm$ 0.018
	CNN	0.981 $\pm$ 0.005	0.959 $\pm$ 0.029	0.968 $\pm$ 0.018	0.979 $\pm$ 0.007	0.979 $\pm$ 0.007
HC-NH	RF	1.000 $\pm$ 0.000	1.000 $\pm$ 0.000	1.000 $\pm$ 0.000	1.000 $\pm$ 0.000	1.000 $\pm$ 0.000
	SVM	0.999 $\pm$ 0.001	0.998 $\pm$ 0.004	0.999 $\pm$ 0.003	0.999 $\pm$ 0.002	0.999 $\pm$ 0.002
	ANN	0.961 $\pm$ 0.011	0.967 $\pm$ 0.014	0.963 $\pm$ 0.008	0.971 $\pm$ 0.005	0.971 $\pm$ 0.005
	CNN	0.992 $\pm$ 0.006	0.983 $\pm$ 0.017	0.987 $\pm$ 0.012	0.990 $\pm$ 0.010	0.990 $\pm$ 0.010
UC-CD	RF	0.973 $\pm$ 0.019	0.971 $\pm$ 0.019	0.972 $\pm$ 0.019	0.972 $\pm$ 0.019	0.972 $\pm$ 0.019
	SVM	0.963 $\pm$ 0.015	0.963 $\pm$ 0.015	0.963 $\pm$ 0.015	0.963 $\pm$ 0.015	0.963 $\pm$ 0.015
	ANN	0.950 $\pm$ 0.023	0.950 $\pm$ 0.022	0.950 $\pm$ 0.023	0.950 $\pm$ 0.022	0.950 $\pm$ 0.022
	CNN	0.963 $\pm$ 0.018	0.960 $\pm$ 0.018	0.961 $\pm$ 0.018	0.961 $\pm$ 0.018	0.961 $\pm$ 0.018

**Fig. 6:**
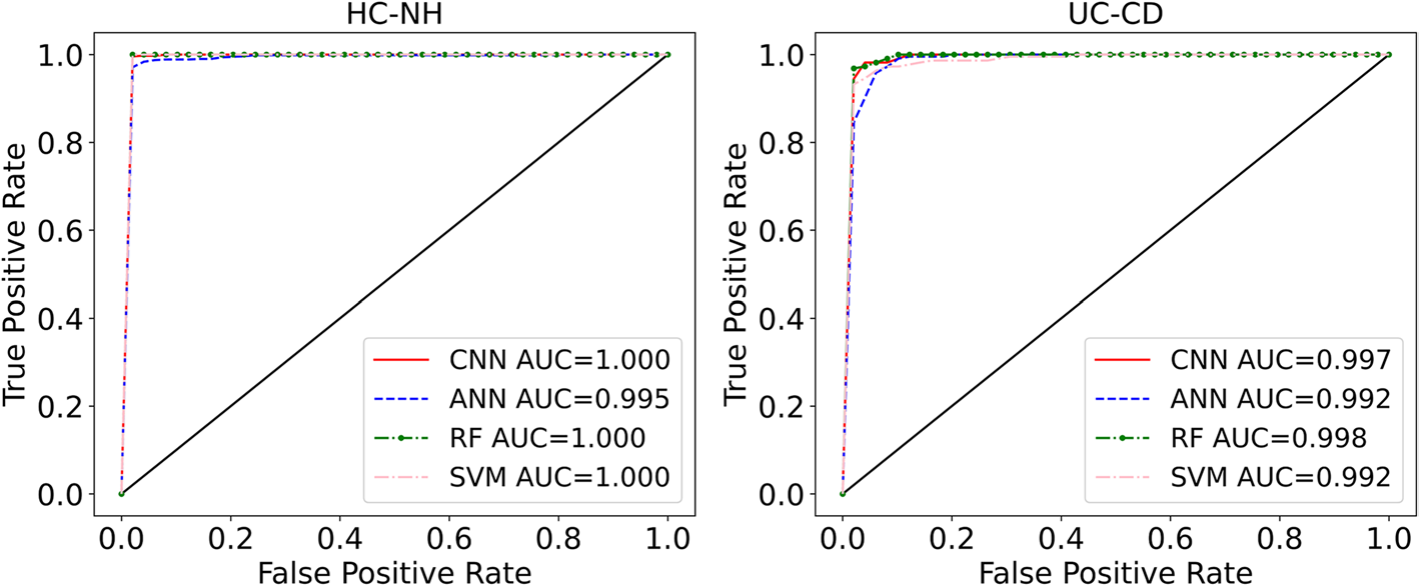
ROC curves with AUC values listed for the binary classification of HC vs. NH and UC vs. CD using the fiber dataset

In the fiber dataset, as with the baseline dataset, misclassifications regarding the HIV cases persisted, likely due to their low abundance in the training datasets. However, the misclassification of the PD cases is no longer observed likely owing to the larger sample size in the fiber dataset compared to the baseline. The highest classification accuracy was achieved in distinguishing HC vs. NH cases, suggesting that ML algorithms can perfectly discriminate between these groups—even in the presence of environmental shifts that affect the gut microbial composition. The ROC curves and AUC values further confirm the strong performacne of the current ML methods, as shown in Fig. 6. As evident, the algorithms almost behave as perfect classifiers for the task HC vs. NH, which is very promising. This suggests that all the four diseases used in this study induce distinct alterations in the microbiome, which are conserved and can be detected by ML algorithms even after fiber treatment. Furthermore, we conducted dimensionality reduction through random projection and PCA and applied machine learning analysis to the fiber dataset using the reduced-dimension data. The models achieved comparable predictive performance for the five conditions, as demonstrated in supplementary section B.

### Visualization

**Fig. 7:**
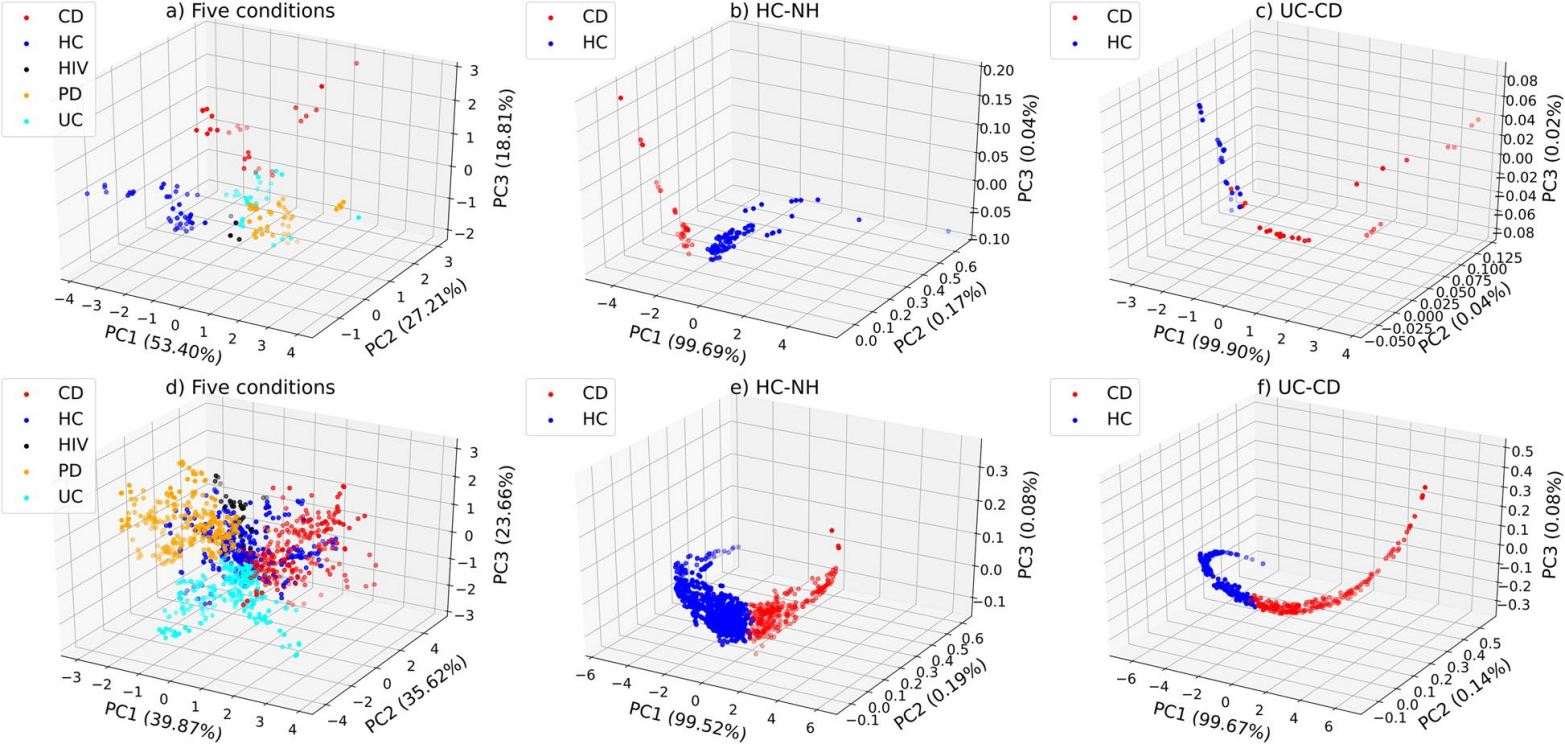
Visualization of the **a**) five conditions **b**) HC vs. NH **c**) UC vs. CD for the baseline dataset, and **d**) five conditions **e**) HC vs. NH **f**) UC vs. CD for the fiber dataset. We extracted the last layer of ANN and applied PCA for the purpose of visualization

To further demonstrate the strength of the ML models, we extracted the values of the last activation layer of ANN and conducted the principal component analysis (PCA) to visualize the data, as shown in Fig. 7. This approach illustrates how supervised learning using neural networks can pave the way for non-linear transformation that facilitate class separation in high-dimensional data. Unlike conventional 2D PCA plots, we used three principal components to generate 3D visualizations as the third principal component contributed meaningfully to class separation—particularly in the five-condition classification task. We observe a clear distinction of different labels for both the baseline and fiber datasets, which aligns with high accuracy values we obtained in Table 1 and Table 2. For instance, in the NH vs. HC classification, for both the baseline and fiber datasets, we observe a clear distinction, aligning with the high AUC values observed for these cases. These distinct clusters suggest that the gut microbiota profile of healthy individuals and those with different disease states can be effectively distinguished through ML—even after environmental shifts (fiber fermentation). These findings highlight the importance of ML-based microbiome analysis as a tool for disease diagnosis, both for conditions that directly affect the gut and those with systemic effects.

## Conclusion

Studies of gut microbiota signatures often target one specific disease or state; however, comparisons of the gut microbiota across different diseases can be challenging due to overlapping pathways that affect the gut. For instance, intestinal inflammation–a strong modifier of the gut microbial community [36] is a common finding in several diseases, including UC, CD, PD, and HIV enteropathy [37]–[38]. Moreover, there are a number of individualized physiological symptoms related to the gut microbiota within diseases that further make such classification difficult. In the current study, different ML algorithms have been applied for the classification of five disease conditions, including PD, UC, CD, HIV, and HC—across three classification tasks . All the genomic data were preprocessed and transformed into OTU forms and then normalized. We used two different datasets: a baseline dataset, where no treatment was administered to patients, and a fiber dataset, which includes both untreated samples and samples following dietary fiber treatment. Our results demonstrate that it is possible to distinguish among the five conditions with accuracies reaching up to 95%, with SVM yielding the best performance among all methods. Although neural networks were included for comparison and visualization purpose, they did not yield significant improvements in classification accuracy compared to other models. Importantly, classifiers achieved near-perfect accuracy in distinguishing HC from NH individuals in both datasets. Additionally, we showed that UC can be distinguished from CD with high accuracy using the OTU-based genomic data—even after fiber treatment. This finding has a major clinical impact because current methods, including invasive diagnostic tools like endoscopic procedures, can fail to distinguish CD from UC in up to 20% of cases [39]. Accurate discrimination between CD and UC is essential for selecting appropriate surgical treatment when needed. Future prospective large cohort studies are required to confirm our proposed method. In the case of PD, current diagnosis is primarily based on clinical symptoms and signs, which are subject to inaccuracy [40]. Brain imaging diagnostic tools are expensive, not widely available, and offer suboptimal sensitivity and specificity [41, 42]. Thus, if future large-scale cohort studies—including patients with PD and Parkinsonism—confirm our finding, then the use of non-invasive stool microbiota can be used as an objective means to diagnose PD accurately, the second most common neurodegenerative disease with an alarming increase in its incidence in western societies [43]. Additionally, as demonstrated in supplementary section B, dimensionality reduction using methods such as PCA and random projection revealed that the data can be linearly separated, with consistent disease-specific patterns. However, it is essential to emphasize that linear separability does not imply causality. Drawing causal inferences from these observations would require further rigorous investigation, such as controlled experiments or causal modeling techniques. Further, it should be noted that our study is a proof-of-concept study, and future studies with much larger sample size and the use of diverse study cohort regarding age, gender, race/ethnicity, and dietary habits are needed to confirm our results and generality of our model for the diagnosis/disease course prediction of these disorders that are associated with gut microbiota dysbiosis.

## Supplementary Information

Supplementary Material 1.

## Data Availability

All the data and python notebook files used in this study are available in the GitHub repository https://github.com/ArezooArdekani/Classifying_diseases_gut_microbiota_ML.
