# Supplementary material for: Machine learning based gut microbiota pattern and response to fiber as a diagnostic tool for chronic inflammatory diseases
Source: BMC Microbiol. 2025 Jun 6;25:353. doi: 10.1186/s12866-025-04072-7 (PMC12143056; doi:10.1186/s12866-025-04072-7)
Supplement: Supplementary file 1 — Supplementary Material 1. [file 12866_2025_4072_MOESM1_ESM.docx]

**A. Prediction based on the augmented baseline data:**

Due to the limited number of HIV cases in our dataset, we augmented the baseline data with an external dataset obtained from Mutlu. et al [1], which included additional HIV cases derived from colonoscopy samples. Furthermore, since the genera present in the new dataset did not exactly match those in our original data, we retained only the common genera, representing approximately 60% of the original dataset’s columns. The abundance of non-overlapping genera was aggregated and added as an additional feature column. The new augmented dataset included 138 cases with 24.59% HC, 24.04% HIV, 21.86% PD, 15.30% UC, and 14.21% CD. We repeated the two classification tasks (five-condition and HC vs. NH) using this augmented dataset. As shown in Table S.1, the models still achieved relatively high accuracy, although there was a slight decrease compared to the original baseline results in Table 1. Additionally, as shown in Figure S.1, the misclassifications regarding the insufficiency of HIV cases have been addressed by increased representation of HIV cases. Similarly, when we increased the number of HIV cases in the fiber dataset, the misclassification rate for HIV cases decreased, as shown in Figure 5. Furthermore, the AUC values for the HC vs. NH classification in the augmented dataset are shown in Figure S.*2*. Remarkably, within our augmented dataset, predictive accuracy consistently exceeded the 0.94.

Table S.1: Classification performances using the augmented baseline dataset.

| **Task** | **Classifier** | Macro precision | Macro Recall | Macro F1 | Micro | Accuracy |
| --- | --- | --- | --- | --- | --- | --- |
| Five conditions | RF | 0.908$\pm$0.047 | 0.906$\pm$0.044 | 0.895$\pm$0.053 | 0.907$\pm$0.048 | 0.907$\pm$0.048 |
|  | SVM | 0.926$\pm$0.046 | 0.933$\pm$0.033 | 0.923$\pm$0.041 | 0.934$\pm$0.033 | 0.934$\pm$0.033 |
|  | ANN | 0.901$\pm$0.044 | 0.899$\pm$0.034 | 0.890$\pm$0.040 | 0.902$\pm$0.037 | 0.902$\pm$0.037 |
| HC-NH | RF | 0.963$\pm$0.020 | 0.844$\pm$0.049 | 0.913$\pm$0.043 | 0.940$\pm$0.032 | 0.940$\pm$0.032 |
|  | SVM | 0.952$\pm$0.051 | 0.966$\pm$0.034 | 0.957$\pm$0.042 | 0.967$\pm$0.032 | 0.967$\pm$0.032 |
|  | ANN | 0.950$\pm$0.048 | 0.962$\pm$0.021 | 0.952$\pm$0.033 | 0.962$\pm$0.028 | 0.962$\pm$0.028 |

**
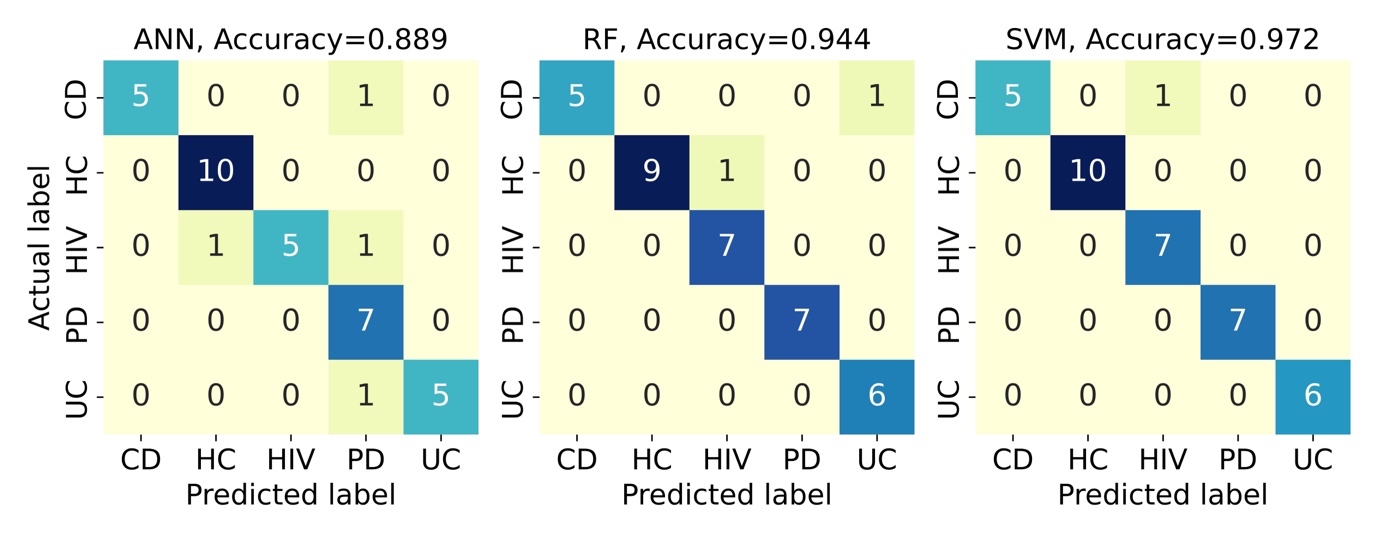
**

Figure S.1: Confusion matrix for the classification of the five conditions using the augmented baseline dataset.

**
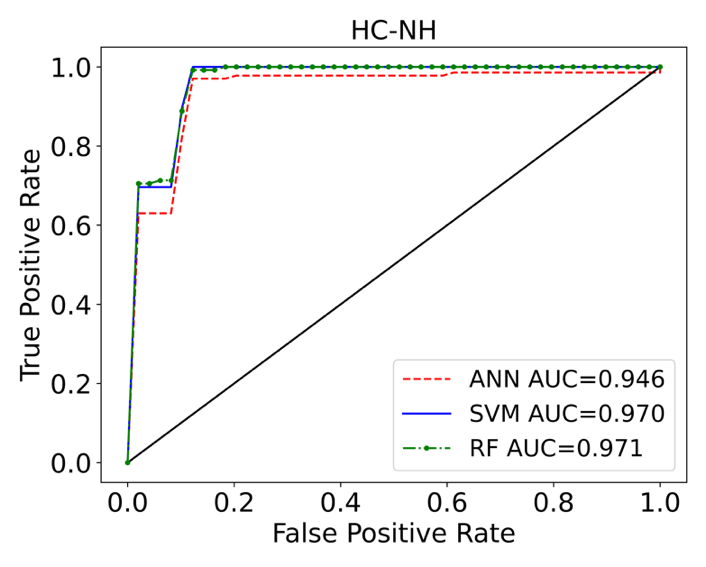
**

Figure S.2: ROC curves with AUC values listed for the binary classification of HC vs. NH using the augmented baseline dataset.

**B. Predictions dimensionality reduction technique:**

**Predictions using random projection technique:**

Random projection is a dimensionality reduction technique commonly used in machine learning and data analysis. It operates by projecting high-dimensional data onto a lower-dimensional subspace using a randomly generated matrix, rather than a predefined transformation. This method is computationally efficient and particularly advantageous for processing large datasets, as it approximately preserves the pairwise distances between data points. In this study, we applied random projection to reduce the dimensionality of the OTU-based data to 30 dimensions. From this reduced space, we selected subsets of 5, 10, and 15 dimensions to evaluate classification performance. We used RF and support vector machine (SVM) models to perform the classification, and the results are summarized in Table S.2 and Table S.3, for the baseline and fiber datasets, respectively. Our results show that classification performance improves as more dimensions are included. Notably, when 15 dimensions are used, the classification metrics closely match those obtained using the full original feature set, consistent with the results reported in Tables 1 and 2.

Table S.2. Classification performances using random projection on the baseline dataset for the classification of five conditions.

| **Random projection** | **Classifier** | Macro precision | Macro Recall | Macro F1 | Micro | Accuracy |
| --- | --- | --- | --- | --- | --- | --- |
| 5 D | RF | 0.763$\pm$0.128 | 0.733$\pm$0.129 | 0.735$\pm$0.130 | 0.833$\pm$0.081 | 0.833$\pm$0.081 |
|  | SVM | 0.841$\pm$0.088 | 0.827$\pm$0.079 | 0.906$\pm$0.028 | 0.906$\pm$0.028 | 0.906$\pm$0.028 |
| 10 D | RF | 0.827$\pm$0.114 | 0.852$\pm$0.083 | 0.852$\pm$0.093 | 0.935$\pm$0.028 | 0.935$\pm$0.028 |
|  | SVM | 0.984$\pm$0.022 | 0.968$\pm$0.048 | 0.971$\pm$0.044 | 0.979$\pm$0.029 | 0.979$\pm$0.029 |
| 15 D | RF | 0.848$\pm$0.111 | 0.823$\pm$0.076 | 0.825$\pm$0.087 | 0.906$\pm$0.018 | 0.906$\pm$0.018 |
|  | SVM | 0.974$\pm$0.023 | 0.942$\pm$0.051 | 0.948$\pm$0.046 | 0.964$\pm$0.032 | 0.964$\pm$0.032 |

Table S.3. Classification performances using random projection on the fiber dataset for the classification of five conditions.

| **Random projection** | **Classifier** | Macro precision | Macro Recall | Macro F1 | Micro | Accuracy |
| --- | --- | --- | --- | --- | --- | --- |
| 5 D | RF | 0.835$\pm$0.059 | 0.786$\pm$0.053 | 0.796$\pm$0.053 | 0.849$\pm$0.035 | 0.849$\pm$0.035 |
|  | SVM | 0.774$\pm$0.049 | 0.762$\pm$0.040 | 0.759$\pm$0.033 | 0.906$\pm$0.028 | 0.906$\pm$0.028 |
| 10 D | RF | 0.931$\pm$0.037 | 0.907$\pm$0.042 | 0.914$\pm$0.040 | 0.930$\pm$0.020 | 0.930$\pm$0.020 |
|  | SVM | 0.931$\pm$0.048 | 0.946$\pm$0.022 | 0.933$\pm$0.034 | 0.951$\pm$0.019 | 0.951$\pm$0.019 |
| 15 D | RF | 0.958$\pm$0.012 | 0.906$\pm$0.055 | 0.923$\pm$0.046 | 0.949$\pm$0.017 | 0.949$\pm$0.017 |
|  | SVM | 0.966$\pm$0.024 | 0.961$\pm$0.024 | 0.961$\pm$0.020 | 0.972$\pm$0.011 | 0.972$\pm$0.011 |

**Predictions using principal component analysis (PCA) technique:**

PCA is a widely used dimensionality reduction technique that transforms the original data into a set of linearly uncorrelated variables known as principal components. These components are ordered based on the amount of variance they explain in the dataset. By selecting and retaining only the top principal components, it is possible to reduce dimensionality while preserving the most informative aspects of the data. Here, we applied PCA to both the baseline and fiber datasets reducing the dimensionality to 5, 10, and 15. As shown in Table S.4 classification performance improved as more components were included. Notably, in the 15-dimensional case using the baseline dataset, the classification accuracy for distinguishing among the five conditions reached as high as 0.97. This level of performance is comparable to that achieved using the full set of genera features, as reported in Table 1, indicating that PCA can effectively reduce data complexity without significant loss of predictive power.

Table S.4. Classification performances using PCA on the baseline dataset for the classification of five conditions.

| **Random projection** | **Classifier** | Macro precision | Macro Recall | Macro F1 | Micro | Accuracy |
| --- | --- | --- | --- | --- | --- | --- |
| 5 D | RF | 0.740$\pm$0.103 | 0.746$\pm$0.084 | 0.736$\pm$0.095 | 0.884$\pm$0.035 | 0.884$\pm$0.035 |
|  | SVM | 0.948$\pm$0.052 | 0.933$\pm$0.060 | 0.934$\pm$0.059 | 0.943$\pm$0.048 | 0.943$\pm$0.048 |
| 10 D | RF | 0.895$\pm$0.096 | 0.882$\pm$0.070 | 0.883$\pm$0.079 | 0.964$\pm$0.001 | 0.964$\pm$0.001 |
|  | SVM | 0.980$\pm$0.017 | 0.959$\pm$0.038 | 0.964$\pm$0.032 | 0.978$\pm$0.018 | 0.978$\pm$0.018 |
| 15 D | RF | 0.978$\pm$0.021 | 0.964$\pm$0.046 | 0.965$\pm$0.042 | 0.971$\pm$0.027 | 0.971$\pm$0.027 |
|  | SVM | 0.980$\pm$0.017 | 0.959$\pm$0.038 | 0.964$\pm$0.032 | 0.978$\pm$0.018 | 0.978$\pm$0.018 |

Similarly, for the fiber dataset, PCA was applied to reduce the data to 5, 10, and 15 dimensions. As shown in Table S.5, classification performance improved progressively as more principal components were included. Notably, when using 15 components, the predictive performance matched that obtained using the full set of original features, as reported in Table 2.

Table S.5. Classification performances using PCA on the fiber dataset for the classification of five conditions.

| **Random projection** | **Classifier** | Macro precision | Macro Recall | Macro F1 | Micro | Accuracy |
| --- | --- | --- | --- | --- | --- | --- |
| 5 D | RF | 0.882$\pm$0.03 | 0.816$\pm$0.077 | 0.832$\pm$0.065 | 0.856$\pm$0.029 | 0.856$\pm$0.029 |
|  | SVM | 0.844$\pm$0.024 | 0.795$\pm$0.043 | 0.810$\pm$0.040 | 0.818$\pm$0.018 | 0.818$\pm$0.018 |
| 10 D | RF | 0.962$\pm$0.017 | 0.930$\pm$0.042 | 0.942$\pm$0.032 | 0.954$\pm$0.042 | 0.954$\pm$0.042 |
|  | SVM | 0.977$\pm$0.008 | 0.965$\pm$0.016 | 0.970$\pm$0.012 | 0.972$\pm$0.009 | 0.972$\pm$0.009 |
| 15 D | RF | 0.972$\pm$0.014 | 0.954$\pm$0.027 | 0.961$\pm$0.021 | 0.966$\pm$0.016 | 0.966$\pm$0.016 |
|  | SVM | 0.984$\pm$0.009 | 0.972$\pm$0.022 | 0.977$\pm$0.016 | 0.981$\pm$0.010 | 0.981$\pm$0.010 |

In our analysis, we observed that the data could be linearly separated using dimensionality reduction techniques such as random projection and PCA, enabling effective ML classification. This linear separability suggests the presence of underlying patterns or relationships within the dataset. However, it is important to emphasize that linear separability does not imply causality. Drawing causal inferences from such observations would require further rigorous investigations, including controlled experiments or causal inference modeling. Our primary goal in highlighting these patterns is to provide insight into the intrinsic structure of the data, which may support improved feature selection and enhance classification performance.
